# Role of Selenium and Vitamins E and B9 in the Alleviation of Bovine Mastitis during the Periparturient Period

**DOI:** 10.3390/antiox11040657

**Published:** 2022-03-29

**Authors:** Muhammad Zahoor Khan, Yulin Ma, Jianxin Xiao, Tianyu Chen, Jiaying Ma, Shuai Liu, Yajing Wang, Adnan Khan, Gibson Maswayi Alugongo, Zhijun Cao

**Affiliations:** 1 Beijing Engineering Technology Research Center of Raw Milk Quality and Safety Control, State Key Laboratory of Animal Nutrition, College of Animal Science and Technology, China Agricultural University, Beijing 100193, China; zahoorcau@cau.edu.cn (M.Z.K.); bs20193040395@cau.edu.cn (Y.M.); xiaojianxin-dairy@cau.edu.cn (J.X.); chentianyu@cau.edu.cn (T.C.); majiaying@cau.edu.cn (J.M.); liushuaicau@cau.edu.cn (S.L.); yajingwang@cau.edu.cn (Y.W.); lb2020270153@cau.edu.cn (G.M.A.); 2 Faculty of Veterinary and Animal Sciences, University of Agriculture, Dera Ismail Khan 29220, Pakistan; 3 Shenzhen Branch, Guangdong Laboratory for Lingnan Modern Agriculture, Genome Analysis Laboratory of the Ministry of Agriculture, Agricultural Genomics Institute at Shenzhen, Chinese Academy of Agricultural Sciences, Shenzhen 518120, China; dr.adnan93@cau.edu.cn

**Keywords:** oxidative stress, periparturient period, bovine mastitis, vitamins, selenium, antioxidants

## Abstract

Mastitis (inflammation of the mammary gland) commonly occurs in dairy cattle during the periparturient period (transition period), in which dairy cattle experience physiological and hormonal changes and severe negative energy balance, followed by oxidative stress. To maintain successful lactation and combat negative energy balance (NEB), excessive fat mobilization occurs, leading to overproduction of reactive oxygen species (ROS). Excessive fat mobilization also increases the concentrations of nonesterified fatty acids (NEFA) and β-hydroxybutyric acid (BHB) during the periparturient period. In addition, the excessive utilization of oxygen by cellular respiration in the mammary causes abnormal production of oxidative stress (OS). OS impairs the immunity and anti-inflammatory efficiency of periparturient dairy cattle, increasing their susceptibility to mastitis. To alleviate oxidative stress and subsequent mastitis, antioxidants are supplemented to dairy cattle from an external source. Extensive studies have been conducted on the supplementation of selenium (Se) and vitamins E and B9 to mitigate mastitis during the transition period in dairy cattle. Altogether, in the current review, we discuss the research development on bovine mastitis and its major causes, with special emphasis on oxidative stress during the transition period. Moreover, we discuss the antioxidant, immunoregulatory, and anti-inflammatory properties of Se and vitamins E and B9 and their role in the control of bovine mastitis in periparturient dairy cattle.

## 1. Introduction

The three weeks before and after calving are known as the periparturient period (transition period) in dairy cattle [1,2]. The periparturient period is characterized by negative energy balance, low dry matter intake, metabolic stress [3], physiological and hormonal changes, increased oxidative stress, immune suppression, and abnormal regulation of inflammation in dairy cattle [4,5,6]. During the transition period, excessive lipid mobilization, physiological changes, oxidative stress, inflammation, and immune dysfunction are key biological function processes that expose dairy cattle to various economic diseases, including mastitis [7,8,9,10].

Dry matter intake and energy demands decrease to maintain successful lactation in dairy cattle. Moreover, more oxygen utilization is required for cellular respiration, leading to oxidative stress [11,12,13]. In addition to fulfilling energy requirements, dairy cattle utilize the fat of their adipose tissues. Excessive fat mobilization may lead to overproduction of reactive oxygen species (ROS) and reactive nitrogen species (RNS) [14]. High concentrations of nonesterified fatty acids (NEFA) [15] and β-hydroxybutyric acid (BHB) are among the critical factors that also provoke ROS overproduction [16]. In addition, a high body condition score (BCS) (body condition score > 3.5/5) is another key factor in determining susceptibility to oxidative stress (OS) during the periparturient phase in dairy cattle [17]. Consistently, it has been documented that excessive loss of BCS causes high concentrations of NEFA and overproduction of OS [18].

**Figure 1:**
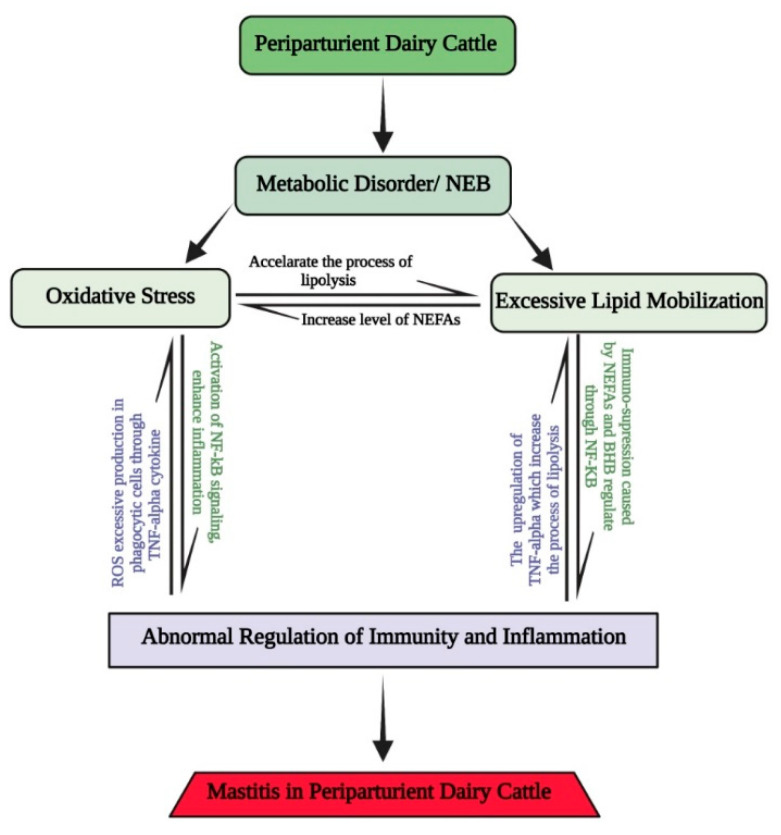
Interrelation of oxidative stress, excessive lipid mobilization, and abnormal immune and inflammation caused by negative energy balance in periparturient dairy cattle. Negative energy balance causes excessive lipid mobilization in periparturient dairy cattle, resulting in increased levels of NEFAs and ROS, leading to oxidative stress. Oxidative stress further causes abnormal regulation of immunity and inflammation, which exposes dairy cattle to mastitis. Abnormal regulation of immunity and inflammation also leads to excessive lipid mobilization and oxidative stress activating overproduction of TNF-α.

A recent study documented that oxidative stress is the primary factor responsible for periparturient diseases in dairy cattle [19]. Similarly, another study reported that the excessive production of ROS causes oxidative stress, which disrupts the immunity and anti-inflammatory functions in dairy cattle during the transition period [20]. Due to immunosuppression, the susceptibility of dairy cattle to mastitis increases during the perinatal phase, as shown in Figure 1 [21,22]. As discussed, negative energy balance and metabolic disorders are key factors that cause oxidative stress during the periparturient period in dairy cattle. Thus, we conclude from the preceding discussion that nutritional management could be one of the most efficient approaches to increase animal antioxidant ability and prevent the oxidative stress that predisposes dairy cattle to mastitis during the transition period [23,24,25,26,27].

Se, vitamin E, and folic acid (B9) have been targeted to improve metabolism, relieve oxidative stress, and enhance the immunity and anti-inflammatory status of perinatal dairy cattle [23,24,25]. Se is an immunomodulator and antioxidant, and its deficiency can predispose dairy cattle to mastitis through activation of the NF-κB/MAPK signaling pathway. Moreover, an increase in oxidative stress has been documented in the mammary gland of Se-deficient mice [28,29]. Similarly, it has been documented that Se and vitamin E deficiency in dairy cattle is associated with increased oxidative stress and somatic cell count (SCC) levels, which may lead to mastitis [30,31,32]. Folic acid (vitamin B9) deficiency may lead to metabolic stress, which eventually causes oxidative stress [2]. Consistently, our research team has documented that folic acid supplementation improves immunity, antioxidant status [23], and glutathione metabolism in dairy cattle [2]. Accordingly, our research team has proved through in vitro and in vivo studies that folic acid treatment significantly downregulates the levels of genes associated with inflammation, reduces cell apoptosis and oxidative stress, and prevents mastitis in dairy cattle [26,27]. Thus, we conclude from the preceding discussion that nutritional management could be one of the most efficient approaches to increase animal antioxidant ability and prevent the oxidative stress that exposes dairy cattle to mastitis during the periparturient period [2,23,25,26,27].

In the current review, we discuss the association of oxidative stress, metabolism, and immunity with mastitis in dairy cattle during the periparturient period. Furthermore, we briefly explain the role of Se, folic acid, and vitamin E as antioxidants, anti-inflammatory agents, and immunomodulators and their role in the prevention of mastitis in dairy during the transition period.

### 1.1. Oxidative Stress and Its Association with Mastitis in Periparturient Dairy Cattle

Under normal physiological conditions, the ability of the antioxidant system is sufficient to neutralize and eliminate ROS produced during metabolic activity. Metabolic changes during pregnancy and calving have been shown to increase ROS generation beyond the necessary level [33]. In addition, a shift in cellular metabolism occurs as the mammary gland prepares for consequent lactation [33]. Large amounts of molecular oxygen are required for aerobic metabolism at the start of copious milk synthesis and secretion. Free radicals are a common byproduct of cellular metabolism, resulting from either the mitochondrial electron transport chain or nicotinamide adenine dinucleotide phosphate (NADPH2) activation [34,35]. As a result, increased rates of ROS production come from the significant increase in oxygen consumption under high metabolic demands. Many studies have documented the excessive ROS generation in the peripheral blood of dairy cattle during the periparturient phase, which overwhelms specific antioxidant defenses, leading to increased oxidative stress [13,16,36].

Excessive production of ROS promotes lipid peroxidation, which leads to oxidative stress, tissue damage, and suppression of glutathione (GSH), a key component of glutathione metabolism in periparturient dairy cattle [37,38]. An imbalance between the generation of ROS and the availability of antioxidant molecules may also lead to oxidative stress, which increases the susceptibility of dairy cattle to various illnesses [34,39]. Moreover, this imbalance disrupts the structure and functions of cellular macromolecules, which predispose periparturient dairy cattle to metabolic diseases [35]. Thus, the normal mechanism to maintain oxidant/antioxidant balance is critical during the periparturient period in dairy cattle [12,40].

Oxidative stress is considered one of the primary variables during the periparturient period, which is associated with the susceptibility of dairy cattle to various diseases such as mammary edema and mastitis [12,13,16,33,41,42,43]. Increased oxidative stress compromises immunity and elevates the susceptibility of dairy cattle to mastitis during the perinatal phase [33,44]. Consistently, Aiken et al. reported that the expression of antioxidant-associated enzymes was significantly low in periparturient dairy cattle [41]. Guan et al. documented higher concentrations of superoxide dismutase (SOD) and glutathione peroxidase (GSH-Px) and a lower concentration of malondialdehyde (MDA) in periparturient cows with low SCC and vice versa [45], which is supported by other published studies [46,47]. OS is associated with abnormal regulation of immunity and inflammation before and after parturition, which has been proven in dairy cattle to coliform mastitis [48,49,50,51,52].

### 1.2. Metabolic Alternation and Its Association with Mastitis in Periparturient Dairy Cattle

It has been well documented that negative energy balance and physiological and metabolic changes aggravate immunosuppression in dairy cattle during the periparturient period [53,54]. Our previous published review highlighted that high milk production stress, hormonal changes, pregnancy burden, and endocrine and metabolic changes may lead to NEB during the periparturient period in dairy cattle [55]. Negative energy balance triggers the increase in lipid fat mobilization, followed by elevation of NEFA and BHB levels, which promotes the abnormal production of ROS and results in oxidative stress in perinatal dairy cattle [45,46,47]. In addition, the authors documented higher levels of NEFA and BHB in high-SCC cows during the periparturient period [45]. Higher levels of NEFA and BHB are associated with oxidative stress, which disrupts the immune system and enhances the inflammatory status, including lower expression of IL-10, increased blood neutrophil-to-lymphocyte ratio (NLR) and platelet-to-lymphocyte ratio (PLR), and higher levels of IL-6, TNF-α, and p-selectin glycoprotein ligand-1 (PSGL-1) in cattle with the high level of SCC [45]. Consequently, higher levels of NEFA and BHB also suppress bovine peripheral blood mononuclear cells (PBMCs), disrupt the function of polymorphonuclear leukocytes (PMNLs), and inhibit the production of interferon-γ, which exposes dairy cattle to mastitis during the periparturient period [56,57,58]. Consistently, other studies have also documented that a high concentration of NEFA may cause severe inhibition of interferon-gamma (IFN-γ) levels, which has antibacterial activity, thus increasing the chances of mammary infections in periparturient dairy cattle [15,23].

### 1.3. Association of Immune System Suppression and Increased Inflammatory Status with Mastitis in Periparturient Dairy Cattle

Hormonal, digestive, and immunological changes in periparturient dairy cattle interfere with immune function, causing immunosuppression and subsequent mastitis [59,60,61]. The periparturient period is regarded as an infection-prone period for dairy cattle due to immunosuppression [62,63]. Moreover, during the first week of lactation, the bactericidal ability of phagocytes and blood PMNLs is significantly reduced, which predisposes dairy cattle to mammary gland infections [64]. Consistently, one study reported that periparturient changes may alter the gene expression profile of neutrophils impacting the immune system of dairy cattle [65]. Furthermore, cells involved in phagocytosis and bacteria encounter activity, and B-cell immunoglobulin synthesis is altered, which may predispose dairy cattle to mastitis during the periparturient period [66]. Moreover, studies have documented that mastitis incidences increase during the perinatal phase because of metabolic disorders and immune system disruption [67,68]. In addition, an increased cortisol level during parturition is also a key factor associated with immunosuppression, which predisposes dairy cattle to mammary gland infections [54].

## 2. Role of Se and Vitamins E and B9 in Overcoming Oxidative Stress, Immune Suppression, Metabolic Imbalance, and Inflammatory Status Associated with Mastitis

### 2.1. Antioxidant Properties of Se and Their Role in Mastitis Alleviation in Periparturient Dairy Cattle

It has been reported that the reduction of hydrogen peroxide and lipid hydroperoxides is catalyzed by plasma glutathione peroxidase (GPx-3), which is a selenocysteine-containing extracellular antioxidant protein [69]. In addition, GPx plays a key role in the antioxidant defense system of dairy cattle [70]. Milk lactoserum has shown undefined antibacterial activity in Se-supplemented dairy cattle [71]. Although the exact mechanism for this antimicrobial activity remains unknown, an increased level of GSH-Px has been found in the blood of dairy cattle treated with Se [72,73]. To prevent oxidative stress, Se has been reported to regulate key antioxidant-associated genes (*TOAX*, *GPX*, *CAT*, *SOD*, and *GSH*) [74]. Consistently, our previously published studies have experimentally proved that superoxide dismutase-1 (SOD1) and catalase (CAT) protect cells from oxidative damage induced by heat stress in bovine granulosa cells [75,76]. Due to its immunological and antioxidant properties, Se has been widely targeted in mastitis control research in dairy cattle.

Mastitis is more common in high-yielding periparturient dairy cattle due to oxidative stress, which promotes changes in the expression of genes associated with proinflammatory factors [41]. Miranda et al. [77] reported that low levels of Se and GPx activity promote oxidative stress in the mammary gland, which is associated with a decrease in mammary epithelial cell numbers. However, in mammary epithelial cells, a balanced level of Se lowers the concentration of hydrogen peroxide [77]. Thus, by lowering hydrogen peroxide levels in mammary epithelial cells, the oxidative status can be eased, resulting in a reduction in apoptotic cells.

In whole blood, GSH-Px activity has been inversely associated with SCC levels in canned milk. This shows that decreased GSH-Px activity during the transition period may expose dairy cattle to mastitis [33]. The reduction in the prevalence of subclinical mastitis in dairy cattle was connected to an increase in GSH-Px activity in the blood following Se treatment [78]. Accordingly, other studies have also verified that Se supplementation positively enhances antioxidant status, improves the GSH-Px level, and decreases the MDA concentration in plasma following decreased SCC in milk [29,79,80,81,82,83,84]. An increased level of MDA is an indication of oxidative stress and subsequent mastitis in periparturient dairy cattle.

Twelve out of twenty-five selenoproteins in animals have shown strong immunological and antioxidant ability [85,86], suggesting that they could be useful options in preventing mastitis in dairy cattle [87]. The level of Se in dairy cattle is associated with the sensitivity of the mammary gland to bacteria [78]. Ali-Vehmas et al. [88] have documented that Se treatment significantly enhances the antibacterial activity, and GSH-Px level of milk. Moreover, the SCC level and mastitis-causing bacteria such as *Escherichia coli* (*E. coli*), *Staphylococcus aureus* (*S. aureus*), *Streptococcus agalactiae*, and *Streptococcus uberis* (*S. uberis*) growth were significantly reduced in response to Se treatment in the milk of dairy cattle [88]. Oxidative stress was restricted by enhancing the level of GSH-Px by selenium in dairy cattle [89,90]. Moreover, it has been documented that Se supplementation reduces the chances of udder infections in dairy cattle [89]. Consequently, a study documented that occurrence of mastitis was spectacularly reduced in response to supplementation of 0.2 mg Se/kg for eight weeks in periparturient dairy cattle [89]. Another study found that 14 out of 36 cows treated with antibiotics during the dry period had mastitis, but only 4 out of 36 cows supplemented with 4 mg of Se during dry milking developed mastitis [84]. Se supplementation enhanced antioxidant ability, followed by an improvement in mammary gland innate and adaptive immunity against mastitis in dairy cattle [71].

It has been reported that *S. aureus* regulates myeloid differentiation factor 88 (MYD88) and utilizes NF-kB signaling to initiate inflammatory changes in the mammary gland after attachment with toll-like receptor (TLR) 2 [91,92]. In a recent study, Wei et al. [93] documented upregulation of MYD88, interleukin-1 beta (IL-1β), TNF-α, pyrin domain-containing protein 3 (NLRP), caspase-recruitment domain (ASC), and caspase-1 in the *S. aureus*-infected macrophages of mice. However, treatment with Se for 90 days significantly reduced the expression of MYD88, IL6, IL-1β, NLRP3, and ASC in the macrophages of mice [93]. In response to any microbial infection and cellular damage, the NLRP3 inflammasome, an important part of innate immunity, regulates caspase-1 activation and the secretion of proinflammatory cytokines IL-1β/IL-18. However, the abnormal regulation of NLRP3 inflammasome by *S. aureus* in the mammary gland is associated with an aberrant inflammatory response [94,95]. The supplementation of selenium significantly downregulated the expression of NLRP3 inflammasome and proinflammatory cytokines IL-1β/IL-18, which activated the abnormal inflammatory response, resulting in mastitis in mice [94,96]. Moreover, Se supplementation suppressed the nuclear transcription factor-kappa B (NF-κB) and mitogen-activated protein kinase (MAPK) signaling pathways that are involved in mastitis progression in mice macrophages [97,98]. This shows that Se supplementation can inhibit the inflammatory changes caused by *S. aureus.*

**Figure 2:**
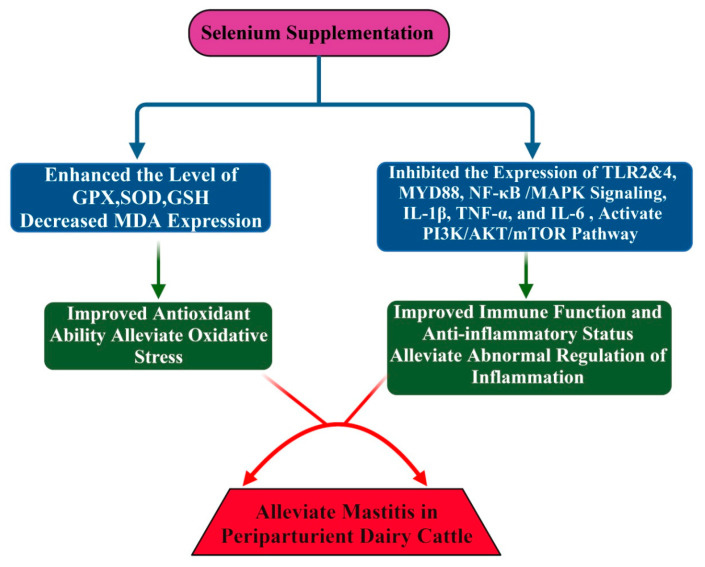
Se enhanced immunity, antioxidant, and anti-inflammatory status in periparturient dairy cattle. Se supplementation significantly improved levels of GPX, GSH, and SOD and reduced levels of MDA during periparturient phase. Phosphorylation levels of NF-κB and MAPKs signaling pathways are inhibited, and expression of IL-1β, IL-6, TLR2, MYD88, and TNF-α is followed by alleviation of inflammation and activation of immunity to relieve mastitis in periparturient dairy cattle.

NF-κB and MAPK signaling play key roles in the initiation of inflammatory changes by promoting cytokine production during mastitis induced by *S. aureus* in the mammary gland [99,100,101]. Consistently, Liu et al. [101] documented that Se supplementation reduced the recruitment of neutrophils and macrophages in mammary epithelial cells. Furthermore, it was documented that TLR2, IL-1β, TNF-α, and IL-6 overexpression levels and increased phosphorylation of NF-κB and MAPKs proteins caused by *S. aureus* were significantly downregulated by Se supplementation in mice [29,96,101,102]. It has been documented that the MerTK plays a key role in regulating PI3K/AKT/mTOR to enhance the anti-inflammatory ability. The PI3K/Akt pathway, upon activation in macrophages by MerTK, can lead to blockage of NF-κB signaling [103]. Furthermore, mediation of the PI3K/AKT/mTOR pathway by MerTK suppressed TLR2-activated immune action and reduced the inflammatory response and oxidative stress in U937 cells [104]. *S. aureus* induced the inflammatory response in the mammary gland of mice by increasing the levels of IL-1β, IL-6, and TNF-α. In addition, the phosphorylation levels of MerTK, PI3K, AKT, and mTOR were decreased in response to *S. aureus* treatment in the mouse mammary gland [105]. The expression of inflammatory cytokines (IL-1β, IL-6, and TNF-α) was reduced, while the phosphorylation levels of MerTK, PI3K, AKT, and mTOR in response to Se treatment were enhanced. These findings further demonstrate that Se could enhance immunity and antioxidant status and reduce the inflammatory response in mammary glands to alleviate mastitis in mice [105]. Jing et al. [106] documented through an in vitro study using the mammary alveolar cell large T antigen (MAC-T) that Se treatment significantly downregulated the expression of genes (*IL1B*, *IRAK4*, *MYD88*, and *SOCS3*) that are associated with mastitis progression in dairy cattle. Moreover, selenium treatment inhibited the activation of PI3K/AKT, MAPK, and NF-κB signaling while accelerating PI3K/Akt/mTOR to promote the anti-inflammatory status in dairy cattle [106]. Furthermore, it has been documented that Se could enhance the expression of IL10, peroxisome-proliferator-activated receptor gamma (PPAR-γ) activity and suppress NF-κB and nitric oxide in the mammary gland of mice caused by *S. aureus*-induced mastitis [107,108]. It was further documented that Se alleviates the oxidative stress and inflammatory status of the mammary gland, which are key factors, leading to the susceptibility of mice to mastitis [107,108]. The role of Se in mastitis alleviation is summarized in Table 1. In addition, the mechanism through which Se alleviates mastitis is explained in Figure 2.

### 2.2. Role of Folic Acid (Vitamin B9) in Mastitis Alleviation of Periparturient Dairy Cattle

In recently published studies, it has been well studied that folic acid plays a key role in the metabolism [2], enhanced immunity, and antioxidant status of periparturient dairy cattle [23]. During the periparturient period, folic acid deficiency may compromise the immunity of dairy cattle [23]. With a key role in immunity and anti-inflammation, folic acid has been targeted in bovine mastitis alleviation in periparturient dairy cattle [27,112,113]. Folic acid supplementation by suppressing MAPK and NF-κB activation maintains the anti-inflammatory status and prevents mastitis [113]. Consistently, a study documented that folic acid supplementation (120 mg/500 kg body weight) for 21 days downregulated all genes associated with immune function and inflammation (*PIM1*, *SOCS3*, *ATP12A*, *KIT,* *LPL* *NFKBIA*, *DUSP4*, *ZC3H12*, *ESPNL*, *TNFAIP3*) [23] that were found to be upregulated in *S. aureus*-induced mastitis during the periparturient period in dairy cattle [114,115]. In addition, in our previous study, we found that folic acid supplementation significantly regulated glutathione metabolism signaling and its related genes (*LAP3*, *GSR*, *G6PD*, *GSTA4*, *GCLC*, *GPX3*, *PGD*, *IDH1*, *GGT1*, *GPX7*, *MGST1*, and *MGST2*) in periparturient dairy cattle [2]. Furthermore, we documented that folic acid could enhance the antioxidant ability of dairy cattle and improve their resistance to mammary gland infection during the periparturient period. Consistently, Mi et al. [26] recently proved experimentally that *S. aureus* induced mastitis in MAC-T cells by downregulating the expression of progenitor renewal associated noncoding RNA (PRANCR). However, in folic-acid-treated MAC-T cells, the expression was higher, showing that folic acid could be the best therapeutic agent in mastitis prevention [26]. Treatment with 5 μg/mL FA significantly reduced apoptosis in Mac-T cells and produced a strong defense against MRSA treatment by improving cytosolic DNA sensing and tight junction signaling [27,116]. They found the upregulation of *ZBP1*, *IRF3*, *IRF7*, and *IFNAR2* within the cytosolic DNA-sensing pathway in FA-treated MAC-T cells. *ZTP1* was reported to be associated with milk SCC [26] and also plays a key role in the activation of anti-pathogen mechanisms and inflammation [117,118]. In addition, the tight junction pathway activation plays a key role against *S. aureus*-induced inflammation in Mac-T cells [116]. Based on published data, we concluded that folic acid supplementation with appropriate dose and duration could be a useful therapeutic agent in mastitis control strategies of dairy cattle during the periparturient phase.

### 2.3. Role of Vitamin E in Periparturient Dairy Cattle Mastitis Alleviation

Vitamin E, a fat-soluble vitamin, protects the cell membrane from the action of lipid peroxidation chain reaction [119,120]. Consistently, a study documented that the polyunsaturated fatty acids in cell membranes of immune cells made them vulnerable to the action of lipid peroxidation by ROS [119,121]. In brief, vitamin E neutralizes peroxyl radicals and prevents polyunsaturated fatty acid oxidation (PUFA). Peroxyl radicals react with α-tocopherol instead of lipid hydroperoxide in the presence of vitamin E, halting the chain reaction of peroxyl radical generation and preventing further oxidation of PUFAs in the membrane [122]. Consistently, it has been found that vitamin E injection alleviates the oxidative stress caused by exposure to aluminum in rats [120]. Additionally, due to its antioxidant property, several studies have documented the importance of vitamin E in regulating immunity and relieving oxidative stress [122,123,124,125,126,127].

The antioxidant properties of vitamin E have been extensively discussed in a previously published review [25]. In addition, vitamin E has been documented to protect damage to bovine mammary endothelial cell barrier integrity induced by pro-oxidants [128]. Consistently, Mokhber-Dezfouli et al. documented that intramuscular injection of vitamin E could reduce MDA expression and lipid peroxidation and improve the antioxidant ability of plasma [129]. Furthermore, it has been established that vitamin E enhances the killing ability of neutrophils and humoral immunity [130,131], while its deficiency causes the abnormal regulation of macrophages and neutrophil functions [132]. In dairy cattle during the periparturient period, vitamin E has been supplemented to alleviate oxidative stress and regulate immunity [133]. Due to its significant role as an antioxidant and immunity regulator, vitamin E has been widely targeted in mastitis mitigation research in periparturient dairy cattle [134].

A considerable decrease in the level of vitamin E has been observed during the transition period in dairy cattle [135]. It has been reported that vitamin E supplementation enhances the anti-inflammatory ability, immunity, and antioxidative ability and reduces mammary infections during the perinatal period in dairy cows [136]. Politis et al. reported that vitamin E deficiency during the periparturient period was significantly associated with oxidation stress and mastitis [137,138]. Consequently, few studies have reported that the supplementation of vitamin E reduced the incidences of mastitis during the transition phase by enhancing the immunity, antioxidant ability, and anti-inflammatory ability in dairy cattle [139,140]. Vitamin E in combination with Se has shown more profitable outcomes in preventing intramammary infections, including mastitis, in dairy cattle [141,142]. Morgante and coauthors [142] also found that somatic cell counts in milk were considerably reduced in response to vitamin E and Se therapy, demonstrating their efficacy in preventing mastitis. Parenteral vitamin E (2100 mg) injections for two weeks before and on calving day have consistently been shown to reduce the incidence of mastitis in dairy cows [142]. Similarly, supplementation of vitamin E (1 g/cow/day) for 30 days prior to calving and up to 60 days after calving dramatically enhanced milk output and reduced the incidence of mastitis in Indian Jersey cattle [143]. According to previously published studies, vitamin E could prevent intramammary infections by environmental pathogens such as *E. coli* and *S. uberis*, which are not generally found in the skin or udder but enter during the periparturient phase when the teat canals in dairy cows are open [144,145,146]. Moreover, it has been documented that vitamin E decreases oxidative stress in the udder and boosts immunity, which is often declined in dairy cows during the perinatal period [144,145,146]. In summary, it has been concluded that dairy cattle experience a severe decrease in the level of vitamin E, which is the key nutrient responsible for immunoregulation to relieve oxidative stress. Oxidative stress is the critical factor associated with abnormal immunoregulation and consequent mastitis in dairy cattle.

## 3. Conclusions

Altogether, we conclude that the periparturient period in dairy cattle is critical and predisposes them to mastitis. Key factors leading to the susceptibility of dairy cattle to mastitis during the transition period are negative energy balance, followed by excessive lipid mobilization, oxidative stress, and consequent abnormal regulation of immunity and inflammation. Dairy cattle experience severe deficiency of some key nutrients (vitamin E, folic acid, and selenium) during the perinatal period, which predisposes them to mastitis. Vitamin (E and folic acid) and Se supplementation positively impact immunoregulation and relieve the oxidative and inflammatory status in dairy cattle during the periparturient phase. Thus, based on the published literature, it is suggested that supplementation of folic acid, selenium, and vitamin E during the transition period could be considered as a therapeutic supplement to alleviate mastitis in dairy cattle.

## Figures and Tables

**Table 1:** Role of Se in improvement of immunity, antioxidant, and anti-inflammatory status to alleviate mastitis.

Treatment	Effects	References
*S. aureus* vs. Se treatment	*S. aureus* induced mastitis in the mammary gland tissue of mice by enhancing the expression of interleukin (IL-1β), IL-6, TNF-α, NF-κB, and MAPK pathways. Se supplementation in mice prevented *S. aureus*-induced mastitis by suppressing the levels of IL-1β, IL-6, TNF-α, NF-κB, and MAPK pathways. Moreover, Se treatment in mice reduced the inflammatory response and alleviated oxidative stress following injuries to mammary gland tissues caused by *S. aureus.*	[101]
*S. aureus* vs. Se treatment	*S. aureus* induced inflammatory changes by upregulating the expression of inflammatory cytokines (IL-1β, IL-6, and TNF-α) and reducing the phosphorylation levels of MerTK, PI3K, AKT, and mTOR.Se treatment reduced the expression of IL-1β, IL-6, and TNF-α and upregulated the phosphorylation levels of MerTK, PI3K, AKT, and mTOR, followed by promoting the anti-inflammatory response and antioxidant status and alleviating mastitis in mice	[105]
*S. aureus* vs. Se treatment	*S. aureus* treatment upregulated the expression of NLRP3, ASC, caspase-1, caspase-1 p20, and pro-IL-1β to enhance the inflammatory response.Se supplementation significantly inhibited the levels of NLRP3, ASC, caspase-1, caspase-1 p20, and pro-IL-1β. This showed that Se treatment prevented *S. aureus*-induced mastitis in mice by suppressing the NLRP3 level.	[94]
*S. aureus* vs. Se treatment	Se treatment inhibited the levels of NLRP3, IL-1β, TNF-α, ASC, and caspase-1 caused by *S. aureus* in the mammary gland of mice. Se supplementation enhanced the antioxidant ability and anti-inflammatory and immune status of mice. In addition, Se treatment prevented mastitis by suppressing NLRP3 inflammasome and NF-κB/ MAPK pathway activation.	[96]
*S. aureus* vs. Se treatment	Se supplementation reduced expression of TLR2 and NF-κB/ MAPK pathway activation caused by *S. aureus* in mice.	[29]
*S. aureus* vs. Se treatment	*S. aureus* induced inflammation in in vivo and primary mouse epithelial cells (MMECs) in vitro. The expression levels of mmu-miR-155, IL-1β, TNF-α, and TLR2 were enhanced. Moreover, the phosphorylation levels of the NF-κB/MAPK signaling pathway were increased in the *S. aureus*-infected mammary epithelial cells of mice. Se significantly inhibited the expression levels of mmu-miR-155, IL-1β, TNF-α, TLR2, and NF-κB/MAPK signaling pathways in the mammary epithelial cells of mice. These findings reveal that Se could prevent mastitis in mice by reducing oxidative stress and the inflammatory response.	[102]
*S. aureus* vs. Se treatment	Se treatment alleviated oxidative stress and inflammatory response; suppressed the expression of IL-1β, TNF-α, ASC, caspase-1, and pro-IL-1β; and inhibited the activation of NLRP3 in *S. aureus*-infected bovine mammary epithelial cells (bMECs).	[109]
*S. aureus* vs. Se treatment	Se supplementation suppressed inflammation induced by *S. aureus* in the mammary gland of mice. Furthermore, the levels of myeloperoxidase (MPO), TLR2, IL-1β, TNF-α, and IL-6 were significantly reduced by Se supplementation in the *S. aureus* mammary gland of mice.	[110]
*S. aureus* vs. Se treatment	Se supplementation suppressed the levels of NF-κB and nitric oxide and enhanced the activation of PPAR-γ activity to prevent *S. aureus*-induced mastitis in mice.	[107]
Se supplementation	Se supplementation significantly improved Se levels in the serum of dairy cattle during the transition phase. Se level is negatively associated with milk SCC and IL6 and positively associated with GSH-Px activity in periparturient dairy cattle	[78]
Se supplementation	Se treatment decreased the level of milk SCC, relieved oxidative stress, and reduced chances of mastitis in periparturient dairy cattle	[111]
Selenite supplementation	Selenite supplementation increased phagocyte recruitment to the infected milk compartment of the udder and enhanced GSH-Px and antibacterial activity of milk lactoserum. In vitro growth of mastitis pathogens was restricted, which shows that Se could be a powerful therapeutic agent for mastitis control.	[88]
Se supplementation	Se supplementation enhanced antioxidant efficiency by promoting GSH-Px activity in Estonian dairy cattle. Furthermore, it was documented that Se-treated cows showed less pathogenic bacteria in their milk.	[89,90]
